# Molecular Context-Dependent Effects Induced by Rett Syndrome-Associated Mutations in MeCP2

**DOI:** 10.3390/biom10111533

**Published:** 2020-11-10

**Authors:** David Ortega-Alarcon, Rafael Claveria-Gimeno, Sonia Vega, Olga C. Jorge-Torres, Manel Esteller, Olga Abian, Adrian Velazquez-Campoy

**Affiliations:** 1 Institute of Biocomputation and Physics of Complex Systems (BIFI), Joint Units IQFR-CSIC-BIFI, and GBsC-CSIC-BIFI, Universidad de Zaragoza, 50018 Zaragoza, Spain; dortega@bifi.es (D.O.-A.); rafacg@bifi.es (R.C.-G.); svega@bifi.es (S.V.); 2 Instituto Aragonés de Ciencias de la Salud (IACS), 50009 Zaragoza, Spain; 3 Instituto de Investigación Sanitaria Aragón (IIS Aragón), 50009 Zaragoza, Spain; 4 Josep Carreras Leukaemia Research Institute (IJC), 08916 Badalona, Spain; ojorge@carrerasresearch.org (O.C.J.-T.); mesteller@carrerasresearch.org (M.E.); 5 Centro de Investigacion Biomedica en Red Cancer (CIBERONC), 28029 Madrid, Spain; 6 Institucio Catalana de Recerca i Estudis Avançats (ICREA), 08010 Barcelona, Spain; 7 Physiological Sciences Department, School of Medicine and Health Sciences, University of Barcelona (UB), l’Hospitalet de Llobregat, 08907 Barcelona, Spain; 8 Centro de Investigación Biomédica en Red en el Área Temática de Enfermedades Hepáticas y Digestivas (CIBERehd), 28029 Madrid, Spain; 9 Departamento de Bioquímica y Biología Molecular y Celular, Universidad de Zaragoza, 50009 Zaragoza, Spain; 10 Fundación ARAID, Gobierno de Aragón, 50009 Zaragoza, Spain

**Keywords:** Methyl-CpG-binding protein 2 (MeCP2), Rett syndrome, intrinsically disordered protein (IDP), protein stability, protein-DNA interaction, isothermal titration calorimetry (ITC)

## Abstract

Methyl-CpG binding protein 2 (MeCP2) is a transcriptional regulator and a chromatin-binding protein involved in neuronal development and maturation. Loss-of-function mutations in MeCP2 result in Rett syndrome (RTT), a neurodevelopmental disorder that is the main cause of mental retardation in females. MeCP2 is an intrinsically disordered protein (IDP) constituted by six domains. Two domains are the main responsible elements for DNA binding (methyl-CpG binding domain, MBD) and recruitment of gene transcription/silencing machinery (transcription repressor domain, TRD). These two domains concentrate most of the RTT-associated mutations. R106W and R133C are associated with severe and mild RTT phenotype, respectively. We have performed a comprehensive characterization of the structural and functional impact of these substitutions at molecular level. Because we have previously shown that the MBD-flanking disordered domains (N-terminal domain, NTD, and intervening domain, ID) exert a considerable influence on the structural and functional features of the MBD (Claveria-Gimeno, R. et al. Sci Rep. **2017**, *7*, 41635), here we report the biophysical study of the influence of the protein scaffold on the structural and functional effect induced by these two RTT-associated mutations. These results represent an example of how a given mutation may show different effects (sometimes opposing effects) depending on the molecular context.

## 1. Introduction

Methyl-CpG binding protein 2 (MeCP2) is an intrinsically disordered protein (IDP) involved in early stages of neuronal development, differentiation, maturation, and synaptic plasticity control [1]. Although it was identified as a methyl-dependent chromatin binding protein and an epigenetic methylation reader, and, therefore, associated to gene silencing, recent evidences suggest it could be considered as a transcriptional regulator whose primary role is recruiting co-repressor complexes to methylated sites and contributing to decreasing transcriptional noise [2].

MeCP2 exhibits a promoter-specific dsDNA interaction required for finely tuning gene transcription, but it also binds massively to heterochromatin when acting as a chromatin architecture remodeling factor. From the initial embryonic development stages, MeCP2 gradually replaces histone 1 as a sort of nucleosomal linker [3,4,5]. The possibility to establish different types of interaction with DNA together with its ability to interact with other many biological partners (RNA, structural and transcriptional proteins, nucleosomal elements) and its central role as an important network interaction hub within gene transcription regulation networks, as well as the additional regulatory level of MeCP2 activity through post-translational modifications are made possible thanks to its modular, dynamic and adaptive structure [6,7].

Abnormal MeCP2 activity leads to disease [2,8,9]. MeCP2 point mutations or deletions causing activity loss are associated with Rett syndrome (RTT). RTT is the main cause of mental retardation in females (1:10,000 births), exhibiting a clinically broad expression phenotype gradation. RTT shares features with other neurological diseases from the autistic spectrum. Importantly, duplication of mecp2 gene results in overexpression of MeCP2 and leads to MeCP2 duplication syndrome (MDS), another much rarer disorder affecting males and, strikingly, sharing phenotypic features with RTT, such as severe intellectual disability and impaired motor function.

Each of the six domains MeCP2 is either completely or partially disordered: N-terminal domain (NTD), methyl binding domain (MBD), intervening domain (ID), transcriptional repression domain (TRD), C-terminal domain α (CTDα), and C-terminal domain β (CTDβ) (Supplemental Figure S1) [3,10]. Because of the importance of the interaction of MeCP2 with the nuclear co-receptor co-repressor (NCoR), an additional NCoR/SMRT interaction domain (NID) is often considered between TRD and CTDα [11]. Most of MeCP2 polypeptide chain (≥60%) lacks well-defined secondary/tertiary structure. Flexible, disordered regions facilitate structural rearrangements necessary for exposing different interaction motifs and adapting to the many interacting partners, as well as the giving rise to the allosteric regulation through which the protein conformational landscape is modulated by ligand binding.

The most important domains are MBD, initially associated with methylated CpG (mCpG) DNA binding, and TRD, associated with transcription repression activities [12,13]. Most RTT-associated mutations are concentrated within these two domains, including missense and nonsense mutations, insertions, duplications, and deletions [14]. Nevertheless, only eight missense and nonsense mutations (R106W, R133C, T158M, R168X, R255X, R270X, R294X and R306C) account for approximately 70% of all mutations in RTT [15]. In particular, R133C, T158M, and R106W (in increasing order for phenotype severity and disease burden) represent 5%, 12%, and 3% of RTT cases [16,17].

MBD is the best characterized domain in MeCP2. MBD structure basically consists of a wedge-shaped structured core containing a 3-stranded anti-parallel β-sheet with an α-helix on the C-terminal side, with two unstructured regions flanking this core [16,17]. MBD is considered to be directly involved in maintaining the global organization of the protein through interactions with other domains through inter-domain coupling [5,18,19]. Mutations in this domain would have an impact on the local and the global stability in MeCP2 [3,18].

In a previous biophysical study of three MeCP2 variants (MBD, and NTD-MBD, and NTD-MBD-ID), we established that the isolated MBD might not be the appropriate construct to study and assay its dsDNA binding features, because the presence of NTD and ID increased considerably the dsDNA binding affinity and the structural stability, besides adding a second, functionally independent dsDNA binding site [20]. Here we report a biophysical study of the structural stability and the dsDNA interaction of mutant variants containing the substitutions R106W and R133C, two main RTT-mutations. These mutations were selected because they consist in an arginine substitution by a bulkier or a smaller residue, they are located in different positions regarding the dsDNA binding interface, and they correspond to different disease severity and burden levels. According to the results presented here, the inclusion of those substitutions into different protein constructions (MBD and NTD-MBD-ID) results in different structural and functional effects, highlighting the importance of selecting an appropriate molecular context (i.e., protein construction) when evaluation mutational effects, and emphasizing, in particular for MeCP2, the potential interdomain interaction in intrinsically disordered proteins [18,19].

## 2. Materials and Methods 

### 2.1. Plasmid Construction

MeCP2 variants from isoform were expressed in *E. coli* using a pET30b plasmid. The different protein variants were obtained by inserting appropriate substitutions: MBD, MBD R106W, MBD R133C, NTD-MBD-ID, NTD-MBD-ID R106W, and NTD-MBD-ID R133C (Supplemental Figure S1). An N-terminal polyhistidine-tag was inserted for quick purification, and it was removed through an inserted PreScission Protease cleavage site. Appropriate expression was assessed by sequencing analysis: Sanger sequencing using a BigDye Terminator v3.1 Cycle Sequencing Kit (Life Technologies, Carlsbad, CA, USA) in an Applied Biosystems 3730/DNA Analyzer (Thermo Fisher Scientific, Waltham, MA, USA).

### 2.2. Protein Expression and Purification

Protein variants (MBD, MBD R106W, MBD R133C, NTD-MBD-ID, NTD-MBD-ID R106W, NTD-MBD-ID R133C) were expressed and purified following identical procedures. Plasmids were transformed into BL21 (DE3) Star *E. coli* strain. Cultures were grown in 150 mL of LB/kanamycin (50 µg/mL) media at 37 °C overnight. Then, 4 L of LB/kanamycin (25 µg/mL) were inoculated (1:100 dilution) and incubated under the same conditions until reaching an OD (λ = 600 nm) of 0.6. Protein expression was induced with 1 mM isopropyl 1-thio-β-D-galactopyranoside (IPTG) at 18 °C overnight. Cells were sonicated in ice and benzonase (Merck-Millipore, Madrid, Spain) was added (20 U/mL) to remove nucleic acids. Proteins were purified using metal affinity chromatography employing a HiTrap TALON column (GE-Healthcare Life Sciences, Barcelona, Spain) with two washing steps: buffer sodium phosphate 50 mM, pH 7, NaCl 300 mM, and buffer sodium phosphate 50 mM, pH 7, NaCl 800 mM. Elution was performed applying an imidazole 10–150 mM elution gradient. Protein purity was evaluated by SDS-PAGE.

The polyhistidine-tag was removed by processing with GST-tagged PreScission Protease in protease buffer (50 mM Tris-HCl, 150 mM NaCl, pH 7.5) at 4 °C for 4 h. Progress of the proteolytic processing was monitored by SDS-PAGE. In the final step the protein was further purified with a combination of two affinity chromatographic steps to remove the polyhistidine-tag (HiTrap TALON column) and the GST-tagged PreScission Protease (GST TALON column, from GE-Healthcare Life Sciences, Barcelona, Spain). Purity and homogeneity were evaluated by SDS-PAGE and size-exclusion chromatography. Storage buffer consisted of Tris 50 mM pH 7.0 and pooled samples were kept at −80 °C. The identity of all proteins was checked by mass spectrometry (4800plus MALDI-TOF/MS, from Applied Biosystems-Thermo Fisher Scientific, Waltham, MA, USA). Potential DNA contamination was always estimated by UV absorption 260/280 ratio. Because a single tryptophan is located in MBD, an extinction coefficient of 11,460 M^−1^ cm^−1^ at 280 nm was employed for all variants, except for the R106W mutants for which a value of 16,960 M^−1^ cm^−1^ was applied.

Stability and binding assays were performed at different pH and buffer conditions (Tris 50 mM pH 7–9, NaCl 0–150 mM; Pipes 50 mM, pH 7; Phosphate 50 mM, pH 7). When needed, buffer exchange was done employing a 3 or 10 kDa-pore size ultrafiltration device (Amicon centrifugal filter, Merck-Millipore, Madrid, Spain) at 4000 rpm and 4 °C.

### 2.3. Double-Stranded DNA

HPLC-purified methylated and unmethylated 45-bp single-stranded DNA (ssDNA) oligomers corresponding to the promoter IV of the mouse brain-derived neurotrophic factor (BDNF) gene [18,19], were purchased from Integrated DNA Technologies. Two complementary pairs of DNA were used for DNA binding assays: forward unmethylated: 5’- GCCATGCCCTGGAACGGAACTCTCCTAATAAAAG-ATGTATCATTT-3’; reverse unmethylated: 5’- AAATGATACATCTTTTATTAGGAGAGTTCCGTTCC-AGGGCATGGC-3’; forward mCpG: 5’- GCCATGCCCTGGAA(5-Me)CGGAACTCTCCTAATAAA-AGATGTATCATTT-3’; reverse mCpG: 5’- AAATGATACATCTTTTATTAGGAGAGTTC(5-Me)CGTT-CCAGGGCATGGC-3’. 

The ssDNA oligonucleotides were dissolved at a concentration of 0.5 mM, mixed at equimolar ratio, and annealed to obtain 45-bp double-stranded DNA (dsDNA) using a Stratagene Mx3005P qPCR real-time thermal cycler (Agilent Technologies, Santa Clara, CA, USA). The thermal annealing profile consisted of: (1) equilibration at 25 °C for 30 s; (2) heating ramp up to 99 °C; (3) equilibration at 99 °C for 1 min; and (4) 3-h cooling process down to 25 °C at a rate of 1 °C/3 min.

### 2.4. Circular Dichroism

Circular dichroism spectra were recorded in a thermostated Chirascan spectrometer (Applied Photophysics, Leatherhead, UK) using a 0.1 cm (far-UV) or 0.4 cm (near-UV) path-length quartz cuvette (Hellma Analytics, Müllheim, Germany) with a bandwidth of 1 nm, a spectral resolution of 0.5 nm, and a response time of 5 s. Temperature was controlled by a Peltier unit and monitored using a temperature probe. The assays were performed in the far-UV (200–260 nm) and the near-UV (250–310 nm) ranges. Protein concentration was set at 10–50 µM, depending on the signal-to-noise ratio.

### 2.5. Fluorescence Spectroscopy

Protein thermal unfolding studies were performed in a Cary Eclipse fluorescence spectrophotometer (Varian—Agilent, Santa Clara, CA, USA) using a protein concentration of 5 µM and a 1 cm path-length quartz cuvette (Hellma Analytics, Müllheim, Germany). The temperature was controlled by a Peltier unit and monitored using a temperature probe, at a heating rate of 1 °C/min. Fluorescence emission spectra were recorded from 300 to 400 nm using an excitation wavelength of 290 nm and a bandwidth of 5 nm. Assays were performed and at the emission wavelength of 330 nm (maximal protein spectral change along the unfolding). A simple two-state unfolding model was considered for analyzing the assays:

(1) $$\begin{array}{c} F\left(T\right)=\frac{\left(A_{N}+B_{N}T\right)+\left(A_{U}+B_{U}T\right)exp\left(-\frac{\Delta G\left(T\right)}{RT}\right)}{1+exp\left(-\frac{\Delta G\left(T\right)}{RT}\right)}\\ \Delta G\left(T\right)=\Delta H\left(T_{m}\right)\left(1-\frac{T}{T_{m}}\right)+\Delta C_{P}\left(T-T_{m}-T\ln\frac{T}{T_{m}}\right) \end{array}$$

where *F*(*T*) is the fluorescence signal at a given absolute temperature *T*, *T_m_* is the unfolding temperature, Δ*H*(*T_m_*) is the unfolding enthalpy (at the *T_m_*), Δ*C_P_* is the unfolding heat capacity, and Δ*G*(*T*) is the stabilization Gibbs energy (which is a temperature function). The adjustable parameters *A_N_*, *B_N_*, *A_U_*, and *B_U_* are instrumental parameters defining the pre- (native) and post-transition (unfolded) regions in the unfolding trace. The stabilizing effect upon dsDNA interaction was assessed performing thermal denaturations of the different proteins (at 5 µM) in the presence of methylated and unmethylated DNA (at 10 µM) under the same conditions.

### 2.6. Isothermal Titration Calorimetry (ITC)

The interaction between the different proteins and dsDNA was studied in an Auto-iTC200 (MicroCal, Malvern-Panalytical, Malvern, UK). dsDNA (50 µM) in the injecting syringe was titrated into protein in the calorimetric cell (3–5 µM). Series of 2 µL-injections of titrant with a time-spacing of 150 s were programmed, maintaining a stirring speed of 750 rpm, and a reference power of 10 μcal/s. The association constant, *K_B_*, and the observed enthalpy of binding, Δ*H_B,obs_*, were estimated through non-linear regression of the experimental data employing a single ligand binding site model (1:1 protein:dsDNA stoichiometry) or a two ligand binding sites model (1:2 protein:dsDNA stoichiometry) implemented in Origin (OriginLab, Northampton, MA, USA) [21,22]. The dissociation constant *K_d_* was calculated as the inverse of *K_B,obs_*, and the binding Gibbs energy and entropy were calculated applying standard well-known relationships: Δ*G* = −*RT* ln*K_B_*, Δ*G* = Δ*H* − *T*Δ*S*.

The number of protons released from or uptaken by the protein-dsDNA complex upon dsDNA binding, Δ*n_H_*, was determined, according to [23,24,25]:

(2) $$\Delta H_{B,obs}=\Delta H+\Delta n_{H}\Delta H_{buffer}$$

where Δ*H* is the buffer-independent binding enthalpy, and Δ*H_buffer_* is the ionization enthalpy of the buffer. Titrations were performed in buffers with different ionization enthalpies (Tris, 11.35 kcal/mol; Pipes, 2.67 kcal/mol; and phosphate, 0.86 kcal/mol) [26] in order to estimate the buffer-independent thermodynamic parameters (Δ*H* and Δ*n_H_*) from linear regression using Equation (2). From Δ*G* and Δ*H*, the buffer-independent binding entropy can be readily calculated. The parameter Δ*n_H_* may be non-zero if ligand binding results in changes in the proton dissociation constant of certain ionizable residues (either in the protein or the ligand) as a consequence of changes in their microenvironment upon complex formation. The association binding constant *K_B_* will be not affected by the buffer ionization as long as the *pK_a_* of the buffer is close to the experimental pH. However, the observed binding enthalpy (and, therefore, the observed entropic contribution) will contain an additional contribution from buffer ionization as indicated above. The experimental strategy allows removing the extrinsic contribution from buffer ionization. Noticeably, Δ*n_H_* has practical utility since it reports the change in binding affinity as a result of a (moderate) change in pH, according to Wyman’s linkage relationships [27]:

(3) $$\Delta n_{H}=-{\left(\frac{\partial \log K_{B}}{\partial pH}\right)}_{P}$$

## 3. Results

### 3.1. Mutation R106W Induces Larger Structural Rearrangements Compared to R133C

RTT-associated MeCP2 variants were expressed in *E. coli* and successfully purified by affinity chromatography, allowing us to study the potential destabilizing impact of the selected mutations on the MBD conformation and its interaction with dsDNA.

Far-UV and near-UV CD spectra were recorded to assess whether R106W and R133C mutations might disrupt MBD secondary and tertiary structure. Being tryptophan bulkier than cysteine (which is even smaller than arginine), and being R106 located buried inside the MBD while R133 is solvent exposed, a larger rearrangement would be expected for R106W mutation. In addition, assays with MBD and NTD-MBD-ID variants were carried out to determine the effect of flanking domains might exert on the destabilizing impact of these RTT-associated mutations.

**Figure 1:**
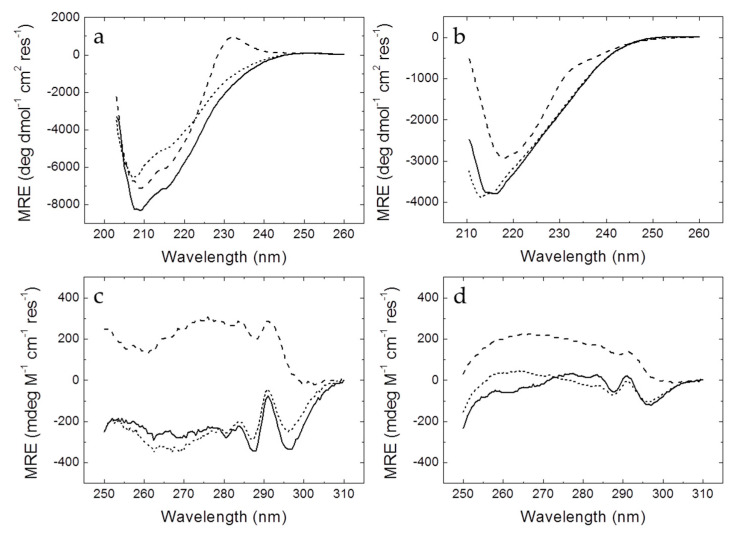
Far-UV (**a**,**b**) and near-UV (**c**,**d**) circular dichroism spectra of wild-type and mutant Methyl-CpG binding protein 2 (MeCP2) methyl-CpG binding domain (MBD) (**a**,**c**) and NTD-MBD-ID (**b**,**d**): wild-type (continuous line), R106W (dashed line), and R133C (dotted line). Spectra were recorded at pH 7.

Far-UV CD spectra of MBD (Figure 1) exhibited two regions typical from β-sheet and random-coil (around 208–210 nm) and α-helix (around 222 nm). MBD mutants showed conserved secondary structure, exhibiting a similar proportion of α-helix and β-sheet to wild-type MBD, but a smaller intensity of the signal could be appreciated, which might be caused by disrupting effects that affected equally to α-helix and β-sheet elements (Figure 1). Furthermore, the MBD R106W mutant showed a positive band around 230 nm typically produced by the presence of an additional tryptophan residue [28]. The far-UV CD spectra of the NTD-MBD-ID mutants exhibited a lower intensity (50% reduction in MRE, molar residue ellipticity) compared to the MBD spectra when normalized by the number of residues, indicating that the flanking domains, NTD and ID, are disordered and hardly contribute to the CD signal. Also, in the presence of NTD and ID the location of the minimum of the R106W CD signal was significantly shifted to larger wavelengths (minimum at 218 nm), while the minimum of R133C CD signal remained at lower wavelengths minimum at 213 nm), showing that these mutations had a considerable impact on NTD-MBD-ID protein secondary structure. Even the wild-type NTD-MBD-ID showed a shifted minimum at larger wavelength (around 215 nm). R106W also exhibited a CD band around 230 nm, compared to the wild type, although it was sensibly smaller than that of MBD R106W. Regarding the near-UV spectra, the R106W mutants exhibit larger alterations than those observed for R133C mutants, whose spectra are quite similar to those of wild-type variants, indicating that in the R106W the environment of the aromatic residues is altered. Similarly, normalization of the spectra with the number of residues results in smaller signal, in agreement with a negligible contribution to the spectra from NTD and ID.

### 3.2. RTT-Associated Mutations Alter Protein Stability and the dsDNA-Induced Stabilization Effect

#### 3.2.1. Stability Changes on MBD Induced by RTT-Associated Mutations

**Figure 2:**
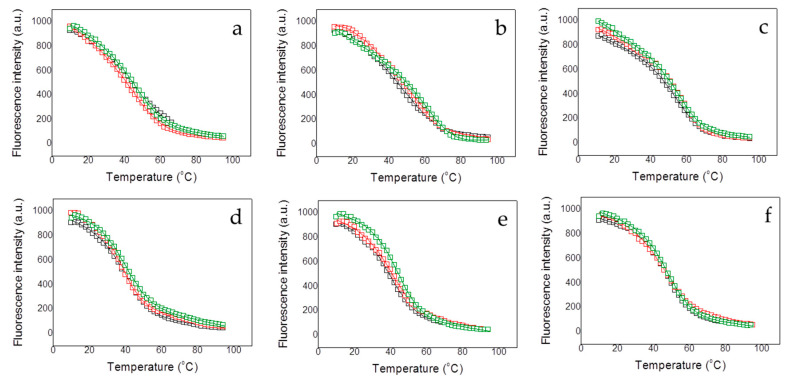
Fluorescence thermal denaturations for the MBD R106W (**a**–**c**), and MBD R133C (**d**–**f**) under different conditions. The influence of the pH was assessed (pH 7, black squares; pH 8, red squares; and pH 9, green squares) (**a**,**d**). The influence of the presence of dsDNA at pH 7 was assessed (absence of dsDNA, black squares; presence of unmethylated CpG-dsDNA, red squares; and presence of methylated mCpG-dsDNA, green squares) at pH 7 and low ionic strength (**b**,**e**) and high ionic strength (**c**,**f**). All unfolding traces could be fitted employing a two-state unfolding model (continuous lines) according to Equation (1).

**Figure 3:**
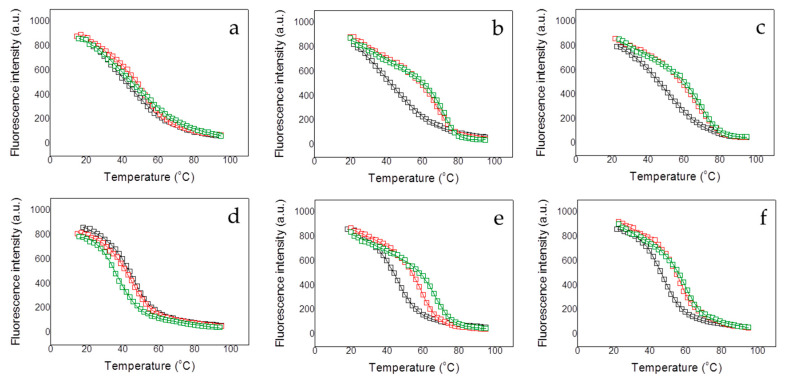
Fluorescence thermal denaturations for the NTD-MBD-ID R106W (**a**–**c**), and NTD-MBD-ID R133C (**d**–**f**) under different conditions. The influence of the pH was assessed (pH 7, black squares; pH 8, red squares; and pH 9, green squares) (**a**,**d**). The influence of the presence of dsDNA at pH 7 was assessed (absence of dsDNA, black squares; presence of unmethylated CpG-dsDNA, red squares; and presence of methylated mCpG-dsDNA, green squares) at pH 7 and low ionic strength (**b**,**e**) and high ionic strength (**c**,**f**). All unfolding traces could be fitted employing a two-state unfolding model (continuous lines) according to Equation (1).

Because of the low content in secondary structure and the small change in the CD signal accompanying the thermal denaturation process, fluorescence spectroscopy was employed for thermal unfolding assays. All protein variants exhibited a well-defined thermal unfolding transition (Figure 2 and Figure 3), which, together with the CD data, indicate that all protein constructs present a well-folded conformation, likely corresponding to the structured region in MBD. 

Except for NTD-MBD-ID R106W, the *T_m_* decreased with pH and increased with ionic strength (Figure 2 and Table 1), as observed with the wild-type variants [20]. This indicates that the unfolding process is coupled to the preferential interaction of protons and salt ions with the folded conformation of the protein variants (i.e., unfolding is accompanied by the release of protons and salt ions).

Each MBD mutation has a different impact on protein stability (Figure 2 and Table 1). Thus, R106W mutation exhibited a stabilizing effect increasing the *T_m_* value in all assayed experimental conditions compared to wild-type MBD. In contrast, R133C mutation did exhibit little impact on protein stability with regard to wild-type MBD, just a slight stabilization in agreement with previous results [18,19]; it seems that this mutation does not affect the protein stability, and, very likely, its deleterious effect might be related to the protein functionality. The stabilizing effect caused by salt ions was similar in both R106W and R133C mutants. MBD R106W and R133C MBD mutants interact preferentially with salt ions in the native state, but very likely salt ions may have an additional charge-screening effect and contribute to the increase stability at high ionic strength by diminishing repulsive interactions between positively charged groups.

One of the most striking results for wild-type MBD was that, at any pH and ionic strength, the structural stability gradually increased with the addition of the disordered domains NTD and ID [20]. The stability of NTD-MBD-ID mutants was also assessed to determine the contribution of those disordered regions, through specific or unspecific effects, to the structural stability (Figure 3 and Table 1). Contrary to wild-type MBD, the addition of NTD and ID may increase or decrease the structural stability of the mutant variant depending on the pH and the ionic strength. For example, at pH 7 and low ionic strength, addition of MBD-flanking domains lowers the stability in the R106W mutant, but raises the stability in the R133C mutant; however, at high ionic strength, addition of MBD-flanking domains lowers the stability of the R106W, while the R133C undergoes no stability change. However, taking NTD-MBD-ID as a reference, both R106W and R133C lowered the stability, in reasonable agreement with previous reported results given the differences in the experimental conditions [18,19]. In general, R133C mutants unfolding parameters are closer to wild-type variants, compared to R106W mutants. Nevertheless, a stabilizing effect caused by R106W mutation can still be observed at higher pH values, indicating a stronger pH dependency of this mutant in the unfolding process.

#### 3.2.2. Stability Changes in RTT-Associated Mutant Proteins Induced by ds-DNA Binding

The interaction of MBD and NTD-MBD-ID mutant variants with dsDNA was indirectly determined by assessing the stabilizing effect induced by unmethylated CpG-dsDNA and methylated mCpG-dsDNA. Thermal denaturations were performed for all MeCP2 protein variants employing the same protocol used for the dsDNA-free variants, in order to determine the apparent thermodynamic parameters for the unfolding of the protein-dsDNA complex (Figure 2 and Figure 3 and Table 2 and Table 3).

In general, dsDNA increased the stability in all variants, as can be observed in the values in *T_m_* compared to those for the dsDNA-free variants, at each experimental condition (Table 2). High ionic strength diminished the extent of the dsDNA stabilization effect (Table 3) through a double mechanism: ionic strength increases protein variants stability and decreases dsDNA binding affinity [20]. MBD R106W is preferentially stabilized by dsDNA, while MBD R133C shows a limited ability to bind dsDNA, because the stabilizing effect induced by dsDNA is very small (Figure 2 and Table 2 and Table 3). A small stabilization effect can be observed for MBD R133C in presence of methylated dsDNA; therefore, MBD R133C might conserve its ability to bind dsDNA, but with a much lower affinity. Wild-type MBD was stabilized by both types of dsDNA, but the stabilizing effect induced by methylated dsDNA was significantly larger. However, MBD R106W showed more similar stabilizing effects for methylated and unmethylated dsDNA.

In the constructs containing the two MBD-flanking domains, NTD-MBD-ID, methylated dsDNA always induced a larger stabilization effect than unmethylated dsDNA (Figure 3 and Table 2 and Table 3), reflecting that the preferential interaction or specificity of MBD towards methylated dsDNA is maintained. Similarly to previous data [21], the MBD-flanking domains, NTD and ID, not only increase (in general) the thermal stability of dsDNA-free MBD mutants, but they also strengthen the stabilizing effect induced by dsDNA binding, and enhance the discriminating power regarding dsDNA methylation as observed by the extent of the stabilization effect. NTD-MBD-ID R106W showed a large stabilizing effect induced by dsDNA, exhibiting a slight difference between both methylated and unmethylated dsDNA effects. NTD-MBD-ID R133C showed an interesting behavior: whereas MBD R133C hardly exhibited a dsDNA-induced stabilizing effect, the addition of the MBD-flanking disordered domains rescued the ability to interact with dsDNA as indicated by the considerable dsDNA-induced stabilization effect. For both MBD and NTD-MBD-ID variants, the substitution R106W induces larger dsDNA stabilization effects compared to the wild-type variant, whereas the substitution R133C induces a smaller stabilization effects. This will be discussed later on.

Although the stabilization effect induced by dsDNA illustrates the fact that MeCP2 mutant constructs are able to interact with dsDNA, this observable cannot be easily employed to measure and quantify binding affinities and other thermodynamic parameters for the interaction in a straightforward manner. The extent of the stabilization effect caused by the presence of dsDNA (Δ*T_m_*) on a given protein conformation depends on the binding affinity, the binding stoichiometry, and the concentration of (free) dsDNA. Furthermore, the binding enthalpy and the binding heat capacity strongly influence the extent of the dsDNA-induced stabilization effect. This makes not possible to estimate binding affinities properly and to establish an affinity ranking based on Δ*T_m_* values. Therefore, further ITC experiments were required to accomplish a biophysical characterization of the interaction between MeCP2 mutants and methylated and unmethylated dsDNA, in order to determine binding affinities and the enthalpic/entropic contributions to the binding.

### 3.3. RTT-Associated Mutations Affect dsDNA Interaction Differently Depending on the MeCP2 Construction

#### 3.3.1. Interaction of MBD Mutants with ds-DNA

**Figure 4:**
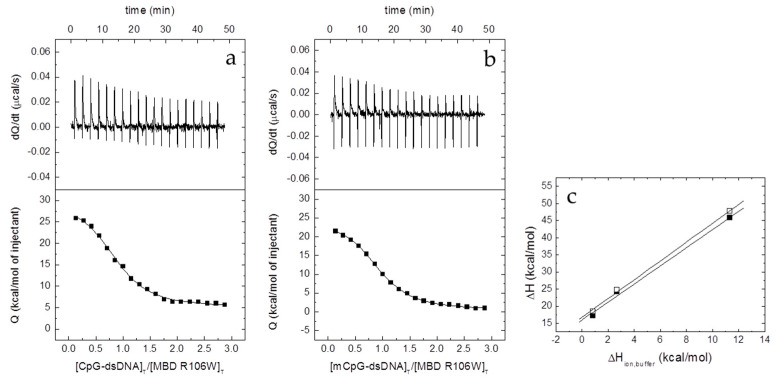
Interaction of the MBD R106W with unmethylated CpG-dsDNA (**a**) and methylated mCpG-dsDNA (**b**) by ITC in Pipes 50 mM, pH 7, 20 °C. Upper plots show the thermogram (raw thermal power as a function of time) and the lower plots show the binding isotherm (ligand-normalized heat effects as a function of the molar ratio). Non-linear least-squares analysis using a single binding site model allowed estimating the observed binding affinity and enthalpy (continuous lines). (**c**) Equation (2) was employed for estimating the buffer-independent binding enthalpy: CpG-dsNA (open squares) and mCpG-dsDNA (closed squares). From linear regression, the intercept with the *y*-axis (observed enthalpy extrapolated at zero buffer ionization enthalpy) provides the buffer-independent interaction enthalpy Δ*H*, and the slope provides Δ*n_H_*.

Contrary to MBD R106W, which shows considerable binding affinity for dsDNA (Figure 4 and Table 4), MBD R133C did not show any interaction with dsDNA under any tested experimental condition (Supplemental Figure S2); this cannot be explained by a loss of structure due to the mutation, because the previous results provided evidence for MBD R133C being properly (partially) folded. The interaction of MBD R106W with dsDNA was characterized by moderate affinity (*K_d_* in the submicromolar range), slightly higher than that of wild-type MBD, and exhibiting a much more favorable entropic contribution to the binding and a much more unfavorable binding enthalpy (Table 4). Opposite to what occurs with wild-type MBD, the formation of the MBD R106W dsDNA complex is accompanied by the uptake of about 2–3 protons from the bulk solution (i.e. at least three ionizable groups are involved in the proton exchange process) (Table 4). Hence, regarding the pH dependency of the binding affinity, increasing the pH in 1 unit would cause a 400-fold decrease in the binding affinity, whereas in the case of wild-type MBD it would cause a 160-fold increase in binding affinity. These results indicated that, although the affinity is comparable or slightly better for MBD R106W, the mode of interaction with dsDNA might be quite different compared to wild-type MBD. In addition, the ability to discriminate between unmethylated and methylated dsDNA was diminished.

#### 3.3.2. Interaction of NTD-MBD-ID Mutants with ds-DNA

**Figure 5:**
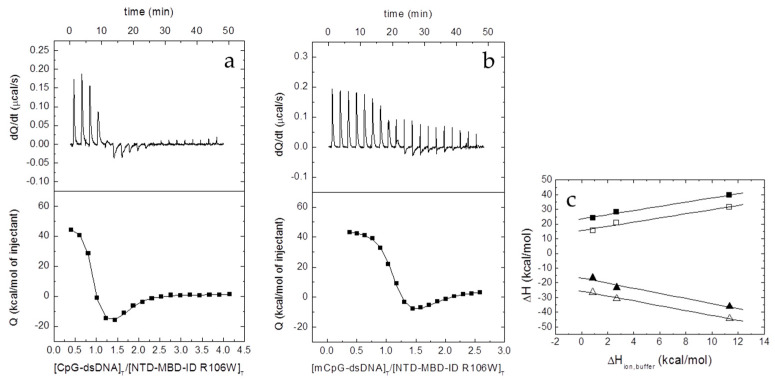
Interaction of the NTD-MBD-ID R106W with unmethylated CpG-dsDNA (**a**) and methylated mCpG-dsDNA (**b**) by ITC in Tris 50 mM, 20 °C, pH 7. Upper plots show the thermogram (raw thermal power as a function of time) and the lower plots show the binding isotherm (ligand-normalized heat effects as a function of the molar ratio). Non-linear least-squares analysis using a two binding sites model allowed estimating the observed binding affinity and enthalpy (continuous lines). (**c**) Equation (2) was employed for estimating the buffer-independent binding enthalpy for both sites (high affinity, squares; low affinity, triangles) for CpG-dsNA (open symbols) and mCpG-dsDNA (closed symbols). From linear regression, the intercept with the *y*-axis (observed enthalpy extrapolated at zero buffer ionization enthalpy) provides the buffer-independent interaction enthalpy Δ*H*, and the slope provides Δ*n_H_*.

**Figure 6:**
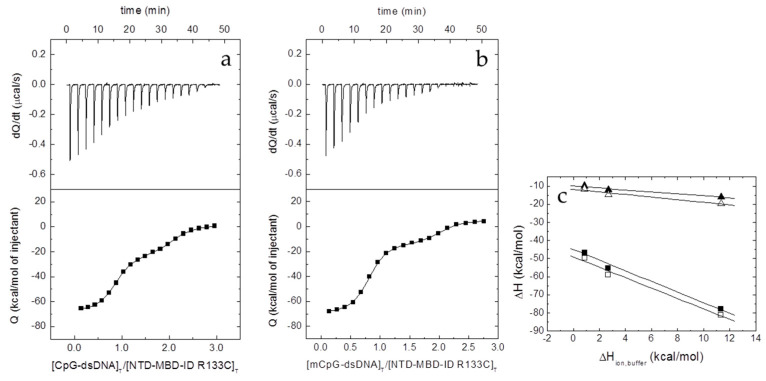
Interaction of the NTD-MBD-ID R133C with unmethylated CpG-dsDNA (**a**) and methylated mCpG-dsDNA (**b**) by ITC in Tris 50 mM, 20 °C, pH 7. Upper plots show the thermogram (raw thermal power as a function of time) and the lower plots show the binding isotherm (ligand-normalized heat effects as a function of the molar ratio). Non-linear least-squares analysis using a two binding sites model allowed estimating the observed binding affinity and enthalpy (continuous lines). (**c**) Equation (2) was employed for estimating the buffer-independent binding enthalpy for both sites (high affinity, squares; low affinity, triangles) for CpG-dsNA (open symbols) and mCpG-dsDNA (closed symbols). From linear regression, the intercept with the *y*-axis (observed enthalpy extrapolated at zero buffer ionization enthalpy) provides the buffer-independent interaction enthalpy Δ*H*, and the slope provides Δ*n_H_*.

It was previously shown that the presence of ID, a small unstructured domain located flanking MBD in its C-terminal position, modified completely the dsDNA interaction: a second interaction site appeared together with a marked increase in the binding affinity of MBD for dsDNA [19,20]. The interaction of the two mutants, NTD-MBD-ID R106W and R133C, could be observed by ITC (Figure 5 and Figure 6, Table 4).

The presence of ID increased the binding affinity of the MBD site in NTD-MBD-ID R106W (taking MBD R106W as a reference), but that increase (3-fold) is small compared to that observed for wild-type (400-fold increase) (Table 4). However, taking NTD-MBD-ID as a reference, the R106W mutation decreased the dsDNA affinity. In NTD-MBD-ID R106W, the binding affinity of the MBD interaction site (high-affinity site) remained in the submicromolar range, comparable to that exhibited by the ID interaction site (low-affinity site), whose binding affinity is only 10-fold lower. Overall, the thermodynamic signature of the interaction was completely opposite to that of the wild type. The R106W mutation strongly disturbs the interaction with dsDNA at the MBD binding site, but also at the ID binding site. In NTD-MBD-ID R133C the addition of the ID domain not only provided an additional dsDNA binding site in ID with a binding affinity in the submicromolar range, but also recovered dsDNA-binding ability in the binding site in MBD with a dramatically increased affinity in the low nanomolar range (Table 4). NTD-MBD-ID R133C did not exhibit large variations in binding affinity regarding the wild type variant, showing similar binding affinities for both the MBD and the ID interaction sites. Strikingly, except the net number of exchanged protons, this mutant exhibited a thermodynamic binding profile similar to that of the wild-type variant. The presence of an additional dsDNA binding site in NTD-MBD-ID variants is responsible, together with the higher affinity, for the much larger dsDNA-induced stabilization effect observed on those variants, compared to the smaller stabilization effects observed for the MBD constructs.

## 4. Discussion

Disordered regions in proteins are characterized by a biased amino acid composition, where residues exhibiting considerable propensity to be exposed to the solvent (polar and charged amino acids) predominate [29]. They may influence protein conformation and function through steric effects, or exerting long-distance attractive or repulsive electrostatic interactions due to their highly polar/charged character, or making contacts with other structured regions affecting the global stability and the dynamics of the protein, as well as modulating the interaction with a binding partner. Thus, even lacking a well-defined structure, disordered regions may contribute to the overall stability of the protein, as it happens in MeCP2. Related to that, we have recently reported that: (1) NTD and ID, the two completely disordered MBD-flanking domains significantly increase the thermal stability of MBD [20]; and (2) the two differentially expressed MeCP2 isoforms (E1 and E2) as a result of differential splicing and differing in just a few amino acids at the N-terminal part of the completely disordered NTD, differ in their thermal stability and functional capabilities [30]. Thus, it may be possible that the conformation and/or the dynamics of MBD is altered by presence of the two disordered flanking domains, resulting in a different stability and different affinity toward binding partners.

**Figure 7:**
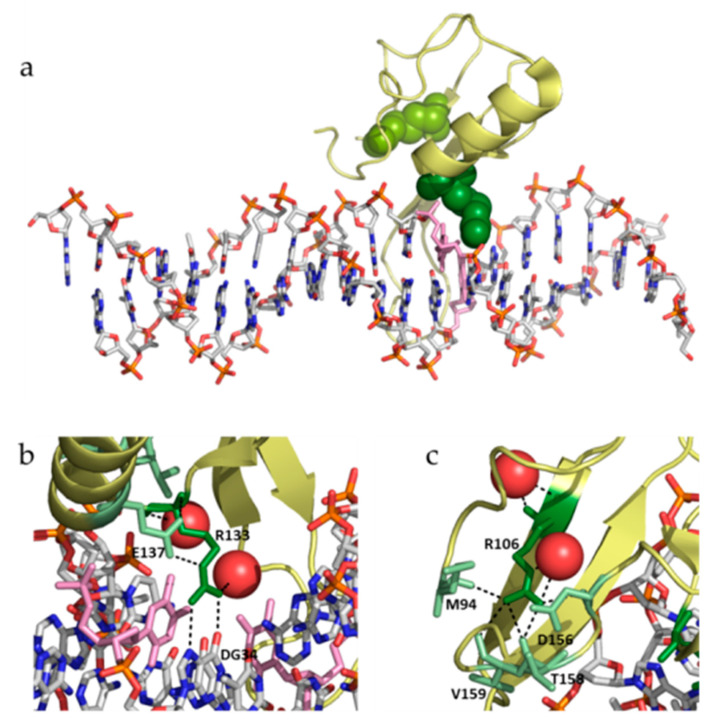
(**a**) Structure of MeCP2 MBD (yellow) in complex with methylated mCpG-dsDNA (CPK view) (pdb id: 3C2I). Residues R106 (light green spheres) and R133 (dark green spheres) are shown. Methylated cytosines are shown as pink sticks (**b**) Close view of R133 (dark green), showing the elements establishing hydrogen bonds with that residue: Glu137 (light green), deoxyguanosine-34 (CPK view), and two water molecules (red spheres). Methylated cytosines are shown in pink sticks. (**c**) Close view of R106 (dark green), showing the elements establishing hydrogen bonds with that residue: Met84, Asp156, T158, V159, and two water molecules (red spheres).

While it is reasonable to expect that the structural and functional impact of point mutations located on structured regions may be predicted with certain reliability, the impact of those located on or close to disordered regions may be more difficult to assess. The two mutations studied in this work, R106W and R133C, are some of the most relevant clinically associated with RTT. They are not located in disordered regions, but in the structured region of the MeCP2 MBD. However, the MBD is very dynamic and susceptible to many environmental factors (pH, temperature, solutes, ligands …), being considered as a key element able to interact with or allosterically regulate the other functional domains [5,18,19]. Both mutations show some similarities and many dissimilarities: (1) both involve the substitution of an arginine residue, but R106W involves the substitution by a bulkier aromatic hydrophobic residue, while R133C involves the substitution by a smaller polar aliphatic residue; (2) R106 is located far from the DNA binding interface, while R133 is located in the DNA binding interface (Figure 7); (3) R106 establishes many interactions with many surrounding residues (in particular, four hydrogen bonding residues: M94, D156, T158, and V159), while R133 interacts with fewer residues (only one hydrogen bonding residue: E137) (Figure 7); and (4) R106 does not interact with DNA, while R133 interacts with DNA through hydrogen bonds and van der Waals contacts (Figure 7). Therefore, the impact of both substitutions is expected to be structurally and functionally different. In fact, if the main rotamers for tryptophan and cysteine are introduced in positions 106 and 133, respectively, all W106 rotamers clash with neighboring residues, while C133 shows no clashes at all, indicating that R106W substitution would result in considerable structural distortion in the vicinity of that position to accommodate such substitution.

As indicated above, the purpose of this work was to gain insight into the relationship between the phenotypic effect and the molecular effect of RTT-associated mutations by assessing the impact of two clinically relevant substitutions in MeCP2 MBD. MBD variants containing the two R106W and R133C mutations were studied regarding their structural stability and dsDNA interaction. In addition, because we have reported before an allosteric coupling between MBD and ID, in which the presence of ID dramatically increased the MBD dsDNA binding affinity and contributed an additional dsDNA binding site, we wanted to address whether the location of those mutations on different scaffolds (MBD or NTD-MBD-ID) would result in different structural and/or functional properties. The experimental strategy consisted of a combination of spectroscopic (CD and fluorescence) and calorimetric (ITC), taking advantage of their strengths and overcoming their limitations. Thus, CD and fluorescence are suitable for gathering coarse-grained structural information, and ITC is the gold-standard for determining binding affinity and providing a complete thermodynamic description of biomolecular interactions. In addition, contrary to other techniques, ITC is appropriate for studying biological interactions with more than one binding site, where the interplay between binding affinity and enthalpy makes easy to observe different binding processes occurring at different locations in a macromolecule.

**Figure 8:**
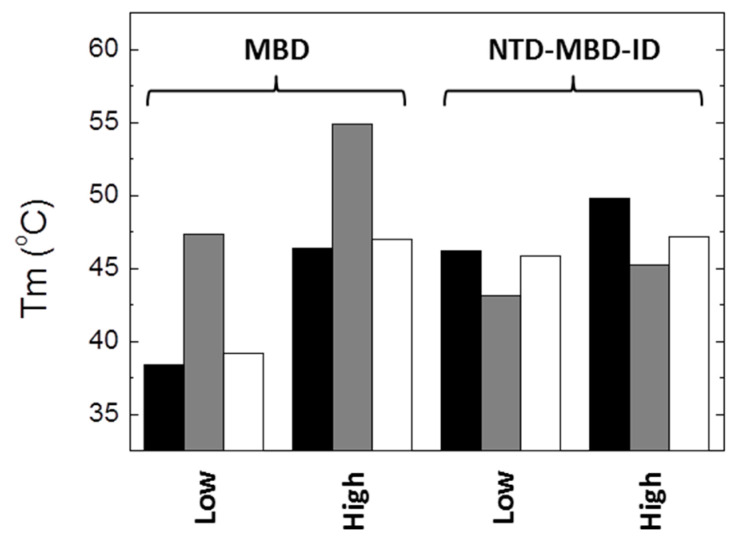
Unfolding temperature for MBD and NTD-MBD-ID variants (wild-type, black bars; R106W, gray bars; R133C, white bars) at low and high ionic strength (NaCl 150 mM).

From the results presented here, it is apparent that the impact of R106W and R133C substitutions on the structural stability and the dsDNA binding capability depends on the molecular context, i.e., the scaffold (MBD or NTD-MBD-ID) in which the substitutions are introduced. Thus, to highlight some of the most important findings: (1) R106W and R133C substitutions increase the thermal stability of MBD, but decrease the thermal stability of NTD-MBD-ID (Figure 8); (2) high ionic strength induces a large stabilization in MBD wild-type and mutants R106W and R133C, maintaining the same stability ranking, but a minor stabilization could be observed for NTD-MBD-ID variants (Figure 8); (3) R106W induces an increase in dsDNA binding affinity in MBD, but a decrease in dsDNA binding affinity in NTD-MBD-ID, compared to their respective wild-type variants; and 4) R133C abolishes dsDNA binding in MBD, but behaves similar to the wild-type variant in NTD-MBD-ID in terms of dsDNA affinity and methyl-dependent discrimination.

The large stabilization observed in NTD-MBD-ID R106W when bound to dsDNA compared to the small stabilization observed for NTD-MBD-ID R133C, taking wild-type NTD-MBD-ID as a reference, may be considered a largely unexpected result (Table 2 and Table 3). The higher dsDNA affinity for the R133C mutant should have induced a larger stabilization extent for that mutant when bound to dsDNA, because the stabilization energy provided by dsDNA binding is equal to +*RT*ln(1+[dsDNA]/*K*_d_). Thus, the extent of the stabilization effect caused by the presence of dsDNA on protein conformation (quantified as increase in stability energy or increase in *T*_m_) depends on the dsDNA binding affinity, the binding stoichiometry, and the concentration of dsDNA. But the binding affinity is dependent on temperature, and, as a consequence, it will change along the thermal denaturation process. The temperature dependency of the binding affinity will be determined by the dsDNA binding enthalpy (and the binding heat capacity), which might not be the same for each interaction (as it occurs for R106W and R133C variants) and will further modulate the overall extent of the ligand-induced stabilization effect. The R106W variant exhibits a strongly endothermic dsDNA binding to the high-affinity site, indicating that, as the temperature starts increasing during the thermal denaturation process, initially the binding affinity and the strength of the complex would increase (according to the van’t Hoff equation) until a temperature in which the binding enthalpy becomes zero (the binding heat capacity is expected to be negative, as found for MBD and NTD-MBD-ID), and then the binding affinity decreases from that temperature. On the contrary, the R133C variant exhibits a strongly exothermic dsDNA binding, and the binding affinity and the strength of the complex would continuously decrease from the beginning of the thermal denaturation process. Therefore, the temperature evolution of *K*_d_ would be different for the two mutants and the stabilization extent would also be different. This is a nice example of two molecules (R106W and R133C variants) binding to a common molecule with different binding affinities, but exerting different stabilization effects: the higher affinity interaction is associated with a smaller stabilization effect.

There are several intriguing facts derived from the experimental results previously shown here. First, how R106W substitution, which is far from the dsDNA binding interface, could affect dsDNA binding and increase its affinity? Of course, even located far from the dsDNA binding interface, the large structural rearrangements resulting from R106W substitution could very likely propagated to distal regions in MBD thanks to its intrinsic structural plasticity. Second, how R133C substitution of a main player in the dsDNA interaction would result in abolishment of interaction for isolated MBD, but almost no effect in NTD-MBD-ID. And third, how these molecular findings can be related to the phenotypic outcomes associated with those mutations: What is the final consequence of R106W substitution at molecular level? Is R106W substitution interfering with the interaction of MeCP2 with other biological partners through the surface residues around R106? What is the final consequence of R133C substitution at molecular level? Is R133C substitution causing an overlooked rearrangement that interferes with other interactions? According to the current classification of RTT mutations, R106W is associated to a severe phenotype, whereas R133C is associated to a mild phenotype [31]. Interestingly, from the evidence gathered in this work, we expect larger functional alterations due to R106W substitution.

## 5. Conclusions

MeCP2 is a potential pharmacological protein target associated with RTT (caused by defective MeCP2 activity) and MDS (caused by excess of MeCP2 activity), two neurological disorders with similar phenotypic features. MeCP2 is mainly involved in neuronal development and maturation, and synaptic plasticity. While the in vivo effect of MeCP2 duplication is still difficult to explain and correlate with cellular events, the in vivo effect of RTT-associated mutations and their connection with molecular and cellular events may be even more challenging. A valuable strategy consists of gathering experimental evidence on the structural and functional impact of those RTT-associated mutations. Isolated MBD, full-length MeCP2, or other constructs have been previously employed as the protein scaffolds for studying those mutations. Because different MeCP2 constructs may behave differently [20,30], a different impact from RTT-associated substitutions might also be expected depending on the molecular context or protein scaffold in which they are introduced. We have provided here evidence for such phenomenon involving R106W and R133C substitutions. This finding underscores the importance of selecting an appropriate protein construction when assessing the effect of a given mutation, being even more important for intrinsically disordered proteins.

Each MeCP2 mutation associated to RTT may cause different perturbations on protein structure, stability and functionality, depending on the disrupted intra- e intermolecular interactions. The environment of the mutation is crucial and strongly influences the potential deleterious impact caused on MeCP2 functionality, thus modulating MeCP2 ability to interact with dsDNA (binding affinity and methyl-dependent discrimination) or other biological partners (RNA and proteins), and further conditioning the ability to undergo functionally-related posttranslational modifications. Mutations can also produce detrimental effects in regions located far apart from them through allosteric coupling. In fact, the ID binding site was fairly compromised in terms of affinity by MBD mutations (Table 4), revealing that the MBD protein structure might be indispensable for ID-dsDNA interaction. Thus, the R106W substitution in NTD-MBD-ID not only affected the MBD interaction site, but its influence was extended to the ID interaction site, causing a non-negligible reduction in binding affinity.

## Figures and Tables

**Table 1:** Thermal stability of the different MeCP2 variants under different conditions.

		*T_m_* (°C)	Δ*H*(*T_m_*) (kcal/mol)
^a^ MBD	pH 7	38.4 ± 0.2	29 ± 1
	pH 8	36.9 ± 0.3	33 ± 2
	pH 9	30.8 ± 0.3	27 ± 1
	pH 7, NaCl 150 mM	46.4 ± 0.4	32 ± 1
^b^ MBD R106W	pH 7	47.3 ± 0.2	21 ± 1
	pH 8	45.8 ± 0.2	20 ± 1
	pH 9	46.7 ± 0.2	23 ± 1
	pH 7, NaCl 150 mM	54.9 ± 0.2	28 ± 1
^b^ MBD R133C	pH 7	39.2 ± 0.2	26 ± 1
	pH 8	38.4 ± 0.3	33 ± 2
	pH 9	38.2 ± 0.2	25 ± 1
	pH 7, NaCl 150 mM	47.0 ± 0.1	29 ± 2
^a^ NTD-MBD-ID	pH 7	46.2 ± 0.2	37 ± 1
	pH 8	45.9 ± 0.3	48 ± 3
	pH 9	45.4 ± 0.2	53 ± 2
	pH 7, NaCl 150 mM	49.8 ± 0.1	38 ± 1
^b^ NTD-MBD-ID R106W	pH 7	43.1 ± 0.3	23 ± 1
	pH 8	52.8 ± 0.3	30 ± 2
	pH 9	54.3 ± 0.3	34 ± 2
	pH 7, NaCl 150 mM	45.2 ± 0.2	37 ± 1
^b^ NTD-MBD-ID-TRD R133C	pH 7	45.8 ± 0.2	33 ± 1
	pH 8	43.8 ± 0.2	31 ± 1
	pH 9	34.5 ± 0.2	30 ± 1
	pH 7, NaCl 150 mM	47.2 ± 0.1	37 ± 2

^a^ Previous work [20]. ^b^ This work.

**Table 2:** Thermal stability of the different MeCP2 variants in the presence of unmethylated (CpG-) and methylated (mCpG-) dsDNA at pH 7.

		*T_m_* (°C)	Δ*H*(*T_m_*) (kcal/mol)
^a^ MBD		38.4 ± 0.3	29 ± 1
	CpG-dsDNA	48.9 ± 0.3	38 ± 2
	mCpG-dsDNA	56.5 ± 0.3	44 ± 2
^b^ MBD R106W		47.3 ± 0.2	21 ± 1
	CpG-dsDNA	60.7 ± 0.2	17 ± 1
	mCpG-dsDNA	64.3 ± 0.2	28 ± 2
^b^ MBD R133C		39.2 ± 0.2	26 ± 1
	CpG-dsDNA	39.4 ± 0.1	24 ± 1
	mCpG-dsDNA	43.5 ± 0.2	29 ± 2
^a^ NTD-MBD-ID		46.2 ± 0.2	37 ± 2
	CpG-dsDNA	64.5 ± 0.1	60 ± 2
	mCpG-dsDNA	71.2 ± 0.2	86 ± 4
^b^ NTD-MBD-ID R106W		43.1 ± 0.2	23 ± 1
	CpG-dsDNA	70.3± 0.2	47 ± 2
	mCpG-dsDNA	72.8 ± 0.2	62 ± 3
^b^ NTD-MBD-ID R133C		45.8 ± 0.2	34 ± 2
	CpG-dsDNA	59.1 ± 0.1	45 ± 2
	mCpG-dsDNA	67.8 ± 0.2	51 ± 3

^a^ Previous work [20]. ^b^ This work.

**Table 3:** Thermal stability of the different MeCP2 variants in the presence of unmethylated (CpG-) and methylated (mCpG-) dsDNA at pH 7 and high ionic strength (NaCl 150 mM).

		*T_m_* (°C)	Δ*H*(*T_m_*) (kcal/mol)
^a^ MBD		46.4 ± 0.4	32 ± 1
	CpG-dsDNA	48.3 ± 0.3	34 ± 2
	mCpG-dsDNA	49.5 ± 0.3	35 ± 2
^b^ MBD R106W		54.9 ± 0.2	27 ± 1
	CpG-dsDNA	57.4 ± 0.2	34 ± 2
	mCpG-dsDNA	58.6 ± 0.2	33 ± 2
^b^ MBD R133C		47.0 ± 0.1	29 ± 1
	CpG-dsDNA	46.6 ± 0.1	28 ± 1
	mCpG-dsDNA	47.7 ± 0.2	29 ± 1
^a^ NTD-MBD-ID		49.8 ± 0.1	38 ± 2
	CpG-dsDNA	65.6 ± 0.2	55 ± 2
	mCpG-dsDNA	66.4 ± 0.2	63 ± 3
^b^ NTD-MBD-ID R106W		45.2 ± 0.1	23 ± 1
	CpG-dsDNA	68.5 ± 0.2	33 ± 2
	mCpG-dsDNA	70.2 ± 0.2	41 ± 2
^b^ NTD-MBD-ID-TRD R133C		47.2 ± 0.1	37 ± 2
	CpG-dsDNA	58.4 ± 0.2	46 ± 2
	mCpG-dsDNA	60.5 ± 0.2	50 ± 2

^a^ Previous work [20]. ^b^ This work.

**Table 4:** Buffer-independent binding parameters for the interaction of the different MeCP2 variants with unmethylated (CpG-) and methylated (mCpG-) dsDNA at pH 7 and 20 °C.

		*K_d_* (nM)	Δ*G* (kcal/mol)	Δ*H* (kcal/mol)	−*T*Δ*S* (kcal/mol)	Δ*n_H_*
^a^ MBD	CpG-DNA	450	−8.5	0.8	−9.3	−2.4
	mCpG-DNA	240	−8.9	1.5	−10.4	−2.1
^b^ MBD R106W	CpG-DNA	110	−9.3	16.7	−26.0	2.7
	mCpG-DNA	95	−9.4	16.0	−25.4	2.6
^b^ NTD-MBD R133C	CpG-DNA	n.i.				
	mCpG-DNA	n.i.				
^a^ NTD-MBD-ID	CpG-DNA	1.9	−11.7	−54.6	42.9	−0.1
		250	−8.9	−7.6	−1.3	−2.9
	mCpG-DNA	0.56	−12.4	−48.4	36.0	−0.1
		62	−9.7	−2.1	−7.6	−1.3
^b^ NTD-MBD-ID R106W	CpG-DNA	27	−10.2	15.6	−25.8	1.4
		320	−8.7	−25.6	16.9	−1.6
	mCpG-DNA	23	−10.2	22.6	−32.8	1.4
		430	−8.5	−16.7	8.2	−1.7
^b^ NTD-MBD-ID R133C	CpG-DNA	2.5	−11.5	−49.1	37.6	−2.9
		83	−9.5	−11.8	2.3	−0.7
	mCpG-DNA	2.1	−11.6	−45.0	33.4	−2.9
		62	−9.7	−10.0	0.3	−0.5

Two dissociation constants *K_d_* (for high-affinity site in MBD and low-affinity site in intervening domain (ID)) are provided for NTD-MBD-ID variants. Relative error in *K_d_* is 15%, absolute error in Δ*G* is 0.1 kcal/mol, absolute error in Δ*H* and −*T*Δ*S* is 0.3 kcal/mol, and absolute error in Δ*n_H_* is 0.2. ^a^ Previous work [20]. ^b^ This work. n.i.: no interaction was observed (at any experimental condition).

## Supplementary Materials

The following are available online at https://www.mdpi.com/2218-273X/10/11/1533/s1, Figure S1: Domain structure of MeCP2, Figure S2: Calorimetric titration of MBD R133C with methylated mCpG-dsDNA.

## References

[1] Ausio J., de Martínez Paz A., Esteller M. (2014). MeCP2: The long trip from a chromatin protein to neurological disorders. Trends Mol. Med..

[2] Tillotson R., Bird A. (2020). The Molecular Basis of MeCP2 Function in the Brain. J. Mol. Biol..

[3] Adkins N.L., Georgel P.T. (2011). MeCP2: Structure and function. Biochem. Cell Biol..

[4] Nan X., Campoy F., Bird A. (1997). MeCP2 Is a Transcriptional Repressor with Abundant Binding Sites in Genomic Chromatin. Cell.

[5] Hansen J.C., Ghosh R.P., Woodcock C.L. (2010). Binding of the Rett syndrome protein, MeCP2, to methylated and unmethylated DNA and chromatin. IUBMB Life.

[6] Guy J., Cheval H., Selfridge J., Bird A. (2011). The Role of MeCP2 in the Brain. Annu. Rev. Cell Dev. Biol..

[7] Claveria-Gimeno R., Abian O., Velazquez-Campoy A., Ausio J. (2016). MeCP2… Nature’s wonder protein or medicine’s most feared one?. Curr. Genet. Med. Rep..

[8] Amir R.E., Veyver I.B.V.D., Wan M., Tran C.Q., Francke U., Zoghbi H.Y. (1999). Rett syndrome is caused by mutations in X-linked MECP2, encoding methyl-CpG-binding protein 2. Nat. Genet..

[9] Neul J.L. (2012). The relationship of Rett syndrome and MECP2 disorders to autism. Dialog. Clin. Neurosci..

[10] Hite K.C., Adams V.H., Hansen J.C. (2009). Recent advances in MeCP2 structure and function. Biochem. Cell Biol..

[11] Kokura K., Kaul S.C., Wadhwa R., Nomura T., Khan M., Shinagawa T., Yasukawa T., Colmenares C., Ishii S. (2001). The Ski Protein Family Is Required for MeCP2-mediated Transcriptional Repression. J. Biol. Chem..

[12] Nan X., Meehan R.R., Bird A. (1993). Dissection of the methyl-CpG binding domain from the chromosomal protein MeCP2. Nucleic Acids Res..

[13] Hall J.A., Georgel P.T. (2007). CHD proteins: A diverse family with strong ties. Biochem. Cell Biol..

[14] Shah R.R., Bird A.P. (2017). MeCP2 mutations: Progress towards understanding and treating Rett syndrome. Genome Med..

[15] Kyle S.M., Vashi N., Justice M.J. (2018). Rett syndrome: A neurological disorder with metabolic components. Open Biol..

[16] Brown K., Selfridge J., Lagger S., Connelly J., De Sousa D., Kerr A., Webb S., Guy J., Merusi C., Koerner M.V. (2016). The molecular basis of variable phenotypic severity among common missense mutations causing Rett syndrome. Hum. Mol. Genet..

[17] Neul J.L., Fang P., Barrish J., Lane J., Caeg E.B., Smith E.O., Zoghbi H.Y., Percy A., Glaze D.G. (2008). Specific mutations in methyl-CpG-binding protein 2 confer different severity in Rett syndrome. Neurology.

[18] Ghosh R.P., Horowitz-Scherer R.A., Nikitina T., Gierasch L.M., Woodcock C.L. (2008). Rett Syndrome-causing Mutations in Human MeCP2 Result in Diverse Structural Changes That Impact Folding and DNA Interactions. J. Biol. Chem..

[19] Ghosh R.P., Nikitina T., Horowitz-Scherer R.A., Gierasch L.M., Uversky V.N., Hite K., Hansen J.C., Woodcock C.L. (2010). Unique Physical Properties and Interactions of the Domains of Methylated DNA Binding Protein 2. Biochemistry.

[20] Clavería-Gimeno R., Lanuza P.M., Morales-Chueca I., Torres O.D.L.C.J., Vega S., Abian O., Esteller M., Velazquez-Campoy A. (2017). The intervening domain from MeCP2 enhances the DNA affinity of the methyl binding domain and provides an independent DNA interaction site. Sci. Rep..

[21] Vega S., Abian O., Velazquez-Campoy A. (2015). A unified framework based on the binding polynomial for characterizing biological systems by isothermal titration calorimetry. Methods.

[22] Freire E., Schön A., Velazquez-Campoy A. (2009). Chapter 5 Isothermal Titration Calorimetry. Methods Enzymol..

[23] Eftink M., Biltonen R.L., Beezer A.E. (1980). Thermodynamics of interacting biological systems. Biological Microcalorimetry.

[24] Hinz H.J., Shiao D.D.F., Sturtevant J.M. (1971). Calorimetric investigation of inhibitor binding to rabbit muscle aldolase. Biochemistry.

[25] Gómez J., Freire E. (1995). Thermodynamic Mapping of the Inhibitor Site of the Aspartic Protease Endothiapepsin. J. Mol. Biol..

[26] Goldberg R.N., Kishore N., Lennen R.M. (2002). Thermodynamic Quantities for the Ionization Reactions of Buffers. J. Phys. Chem. Ref. Data.

[27] Wyman J. (1964). Linked Functions and Reciprocal Effects in Hemoglobin: A Second Look. Adv. Protein Chem..

[28] Vuilleumier S., Sancho J., Loewenthal R., Fersht A.R. (1993). Circular dichroism studies of barnase and its mutants: Characterization of the contribution of aromatic side chains. Biochemistry.

[29] Uversky V.N. (2013). Unusual biophysics of intrinsically disordered proteins. Biochim. Biophys. Acta Proteins Proteom..

[30] De Paz A.M., Khajavi L., Martin H., Claveria-Gimeno R., Dieck S.T., Cheema M.S., Sanchez-Mut J.V., Moksa M.M., Carles A., Brodie N.I. (2019). MeCP2-E1 isoform is a dynamically expressed, weakly DNA-bound protein with different protein and DNA interactions compared to MeCP2-E2. Epigenetics Chromatin.

[31] Cuddapah V.A., Pillai R.B., Shekar K.V., Lane J.B., Motil K.J., Skinner S.A., Tarquinio D.C., Glaze D.G., McGwin G., Kaufmann W.E. (2014). Methyl-CpG-binding protein 2 (MECP2) mutation type is associated with disease severity in Rett syndrome. J. Med. Genet..

