# Supplementary material for: Molecular Context-Dependent Effects Induced by Rett Syndrome-Associated Mutations in MeCP2
Source: Biomolecules. 2020 Nov 10;10(11):1533. doi: 10.3390/biom10111533 (PMC7696773; doi:10.3390/biom10111533)
Supplement: Supplementary file 1 [file biomolecules-10-01533-s001.pdf]

SUPPLEMENTARY MATERIALS

Molecular Context-Dependent Effects Induced by Rett Syndrome-Associated Mutations in MeCP2

David Ortega-Alarcon, Rafael Claveria-Gimeno, Sonia Vega, Olga C. Jorge-Torres, Manel Esteller, Olga Abian, and Adrian Velazquez-Campoy

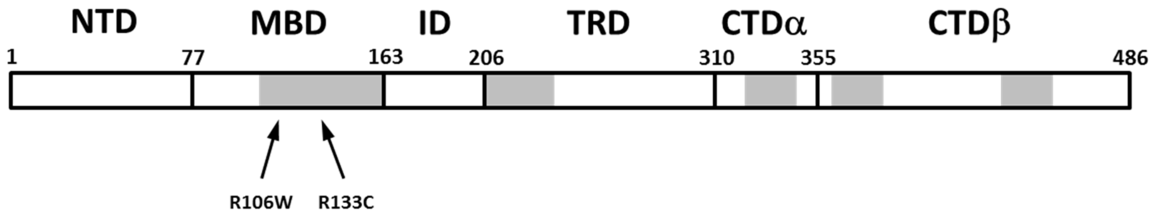

Figure S1. Domain structure in MeCP2. Schematic depiction of the domain organization in MeCP2. Structured regions are shown in grey. Arrows indicate the location of the two Rett syndrome associated mutations studied in this work, while the numbers indicate the initial residue for each domain.

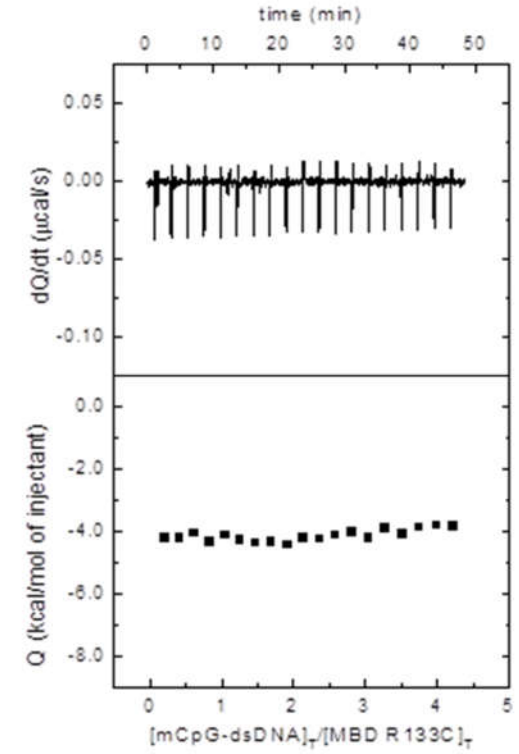

Figure S2. Calorimetric titration for methylated mCpG-dsDNA titrated into MBD R133C. Experiment performed in Pipes 50 mM, pH 7, 20 °C. Upper plot shows the thermogram (raw thermal power as a function of time) and the lower plot shows the binding isotherm (ligand-normalized heat effects as a function of the molar ratio). No interaction was observed, as the heat effects were similar. Changes in experimental conditions (selecting buffers with different ionization enthalpy and varying temperature) did not result in observable interaction.
